# Novel approaches to define responders to interventional treatment in hypertension: insights from the SPYRAL HTN-OFF and HTN-ON MED trials

**DOI:** 10.1038/s41440-024-01949-4

**Published:** 2024-11-14

**Authors:** Roland E. Schmieder, Douglas A. Hettrick, Michael Böhm, David E. Kandzari, Kazuomi Kario, Felix Mahfoud, Konstantinos Tsioufis, Michael A. Weber, Murray D. Esler, Raymond R. Townsend

**Affiliations:** 1 University Hospital Erlangen, Erlangen, Germany; 2 Medtronic, Santa Rosa, CA USA; 3 Universitätsklinikum des Saarlandes, Saarland University, Homburg, Germany; 4 Piedmont Heart Institute, Atlanta, GA USA; 5 Department of Cardiovascular Medicine, Jichi Medical University School of Medicine, Tochigi, Japan; 6 Department of Cardiology, University Heart Center, University Hospital Basel, Switzerland. Cardiovascular Research Institute Basel (CRIB), University Heart Center, University Hospital Basel, Basel, Switzerland; 7 National and Kapodistrian University of Athens, Hippocratio Hospital, Athens, Greece; 8 Department of Medicine, SUNY Downstate College of Medicine, New York, NY USA; 9 Human Neurotransmitter Laboratory, Baker Heart and Diabetes Institute, Melbourne, VIC Australia; 10 Perelman School of Medicine, University of Pennsylvania, Philadelphia, PA USA

**Keywords:** Renal denervation, Responders, Blood pressure variability

## Abstract

Multiple sham-controlled clinical trials have demonstrated significant reductions in both office and 24-h blood pressure (BP) following radiofrequency renal denervation (RDN) in the uncontrolled hypertension population. Notably, the blood pressure response varies widely within individual participants, thus showing a clinical need to identify potential RDN “responders” prior to the procedure. Despite multiple analytic efforts, no single parameter, aside from baseline blood pressure, has been consistently associated with BP reduction following RDN. However, this failure may be due to limitations in empiric definitions of responders. Indeed, commonly applied responder definitions based on the difference between two point-in-time BP measurements are fraught due to visit-to-visit variability in office and 24-h blood pressure endpoints. Several factors should be considered to develop a more clinically useful operational definition of procedural response including relative changes in office and 24-h BP, consideration of the temporal response to RDN, as well as adjustment for baseline BP. The current evidence may provide incentives for future expert consensus to precisely define responders to hypertension treatments.

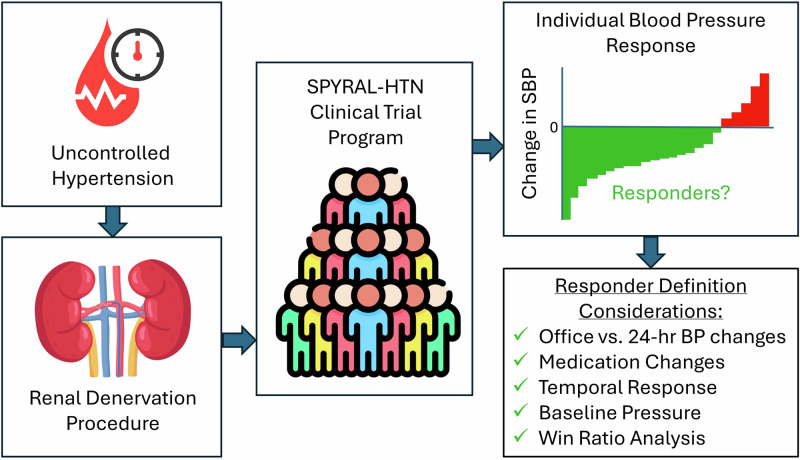

Catheter based renal denervation (RDN) is a treatment option for hypertension uncontrolled by lifestyle modifications and antihypertensive medication. Results from multiple sham-controlled clinical trials demonstrate significant reductions in both office and 24-h blood pressure (BP) following RDN [1–6]. However, the vast size of the uncontrolled hypertension population [7] has shifted clinical focus towards identification of populations that may benefit the most from RDN. Indeed, contemporary clinical consensus statements and position papers suggest that identification of so-called “responders” is one of the most pressing unmet clinical needs for RDN [8–11]. However, this clinically intuitive term is scientifically not well defined. Typically, responders are identified as treated subjects achieving a particular empirically defined threshold of reduction of office systolic BP reduction (for example 10 mmHg) at a single arbitrarily chosen follow-up time point. Multiple parameters associated with increased basal sympathetic activity, such as heart rate [12], plasma renin activity [13], and body mass index [14], as well as indices associated with lower arterial stiffness, including pulse wave velocity [15, 16], central pulse pressure [17], augmentation index [18] and aortic distensibility [19] have been identified as potential markers of procedural response. Likewise, investigation of genetic markers has not indicated that genetic variants or combinations of them could help to identify predictors of BP response after RDN [20]. Applying these definitions, the only clinical variable consistently associated with BP response to date has been severity of baseline BP [21].

Previously, we speculated that this lack of consistency in response predictors was due to in part to the impact of visit-to-visit BP variability on operational definitions of responders based on single-point-in-time measurements [22]. This review leverages available patient level data from recent radiofrequency RDN clinical trials in patients with uncontrolled hypertension, as well as other recently reported clinical data, to appraise the limitations of current definitions and suggest alternative approaches to more accurately designate treatment “responders”.

## Limitations of operational definitions of procedural responders

**Fig. 1:**
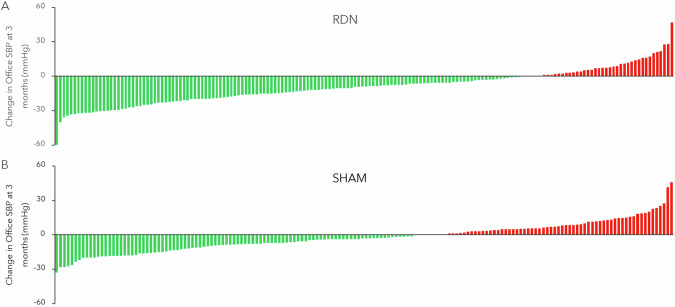
Changes in office SBP to 3 months in the SPYRAL HTN-OFF Med Pivotal trial for the RDN (**A**, *N* = 150) and sham control (**B**, *N* = 134) groups [4]. Green bars indicate patients with a decrease in office systolic blood pressure increase between single-point-in-time measurements at baseline and the 3-month primary endpoint, while red bars indicate patients with an increase

Prospectively powered sham controlled clinical trials for RDN were appropriately designed to detect a BP response within a specific population [23]. However, these methodologies were not necessarily intended to identify response on an individual level. Changes in office and 24-h mean BP between groups following RDN were invariably determined by the difference between two single-point-in-time measurements performed at baseline and at the primary follow up several months later. Responders have been retrospectively identified from these data sets by statistically comparing variables of interest between subgroups of RDN-treated patients with BP reduction above or below a certain threshold, as demonstrated for the SPYRAL HTN-OFF MED trial in Fig. 1A. However, this definition incorrectly assumes that each patient’s BP would have remained constant between measurements. Indeed, SBP did not remain constant in the untreated sham group as shown in Fig. 1B. Note that similar variability in individual BP response has also been shown in drug trials [24]. Hence, data from sham control groups in randomized control trials infer that single-point-in-time measurement cannot accurately identify an individual patient’s response in the RDN group.

**Fig. 2:**
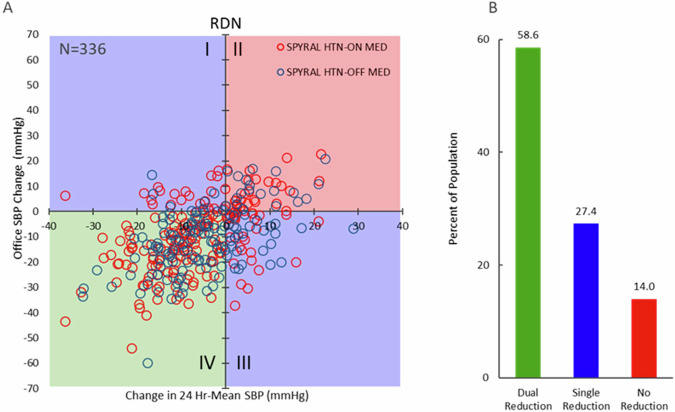
**A** Patient level relationship between the change from baseline in 24-h versus office systolic blood pressure in the SPYRAL HTN-OFF (*N* = 144, blue circles) and -ON MED (*N* = 192, red circles) trials at the primary follow up. Green box (quadrant IV) indicates patients with reductions in both office and 24-h SBP (dual reduction); red box (quadrant II) indicates patients with no reduction in either office or 24-h SBP; blue boxes (quadrants I and III) indication with a reduction in either office or 24-h SBP (single reduction). **B** Proportion of patients in both trials meeting the dual reduction, single reduction, or no reduction classifications (*P* < 0.01; Cochran–Mantel–Haenszel test)

Responders are often classified based on office BP, but can also be independently identified via changes in 24-h mean systolic BP. However, office and 24-h BP are only very loosely correlated with each other within an individual patient [25]. Graphical comparison of the reduction in office SBP plotted against the reduction in 24-h SBP following RDN from the SPYRAL HTN-ON and -OFF MED trials at the primary follow up, as shown in Fig. 2, demonstrates incomplete agreement on responders for office vs 24-h SBP reduction. According to conventional descriptions, patients represented in quadrants II and IV would be classified as non-responders and responders, respectively, since their BP changes are consistent for office and 24-h SBP changes, although large numeric differences in the magnitude of BP changes can be observed within quadrants II and IV. However, the subset of patients (27% of all treated patients) represented in quadrants I and III defy consistent classification, since the BP changes for office and 24-h SBP occur in opposite directions making these patients simultaneously both responders and non-responders. These data suggest that a combined analysis including both changes in office and in 24-h SBP should be considered when defining “responders” to procedural interventions and, likewise, to antihypertensive drugs. Such a combination might increase specificity for accurately identifying a true response but would also reduce sensitivity given the variability in both office and 24-h BP, as further discussed below.

In addition to this graphical approach, a generalized pairwise comparison approach, such as win ratio analysis [26] would further allow hierarchical consideration of multiple variables contributing to a clinically meaningful response [27, 28]. Considering the substantial and intriguing discrepancies between office and 24-hour BP changes, a primary criterion of at least 5 mmHg reduction in 24-h SBP, followed by a secondary criterion of at least 10 mmHg reduction in office SBP could define a “win” (success) for the treatment. A decrease of antihypertensive medication could further be applied as a third win criteria since drug changes also occur. Such a win ratio analysis has been applied in prospective randomized sham controlled RCTs [28]. Additional parameters besides blood pressure changes, such as changes in heart rate [12], arterial mechanical properties [15], or retinal capillary perfusion [29] could also be considered as part of a hierarchical construct.

## Visit-to-visit blood pressure variability and responder identification

**Fig. 3:**
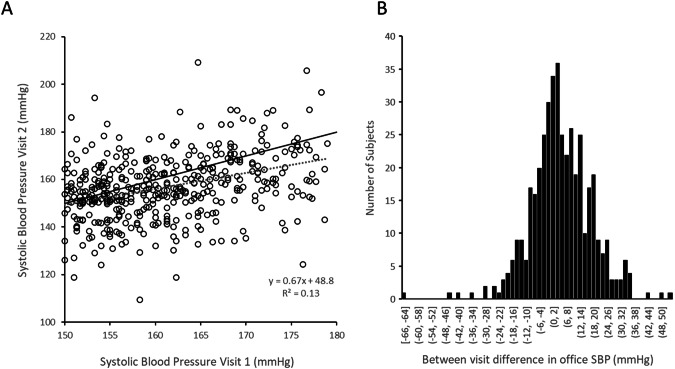
Sub analysis of office SBP in patients enrolled but not randomized in the SPYRAL HTN-ON MED trial (*n* = 434). **A** The mean magnitude of the difference in blood pressure between screening visits 1 and 2 was ±10.7 mmHg. Black solid line: identity; dashed line: linear regression fit. **B** Histogram of between visit difference in office systolic blood pressure

Both office and 24-h BP exhibit substantial visit-to-visit within patient variability, thus complicating identification of individual therapy responders using definitions based on single- point-in-time measurements. A sub analysis of 434 patients enrolled but not randomized in the SPYRAL HTN-ON MED trial compared office SBP between 2 consecutive trial screening visits with no change in medication regimen prior to randomization and RDN treatment (Fig. 3). The mean difference between visits was ±10.7 mmHg the data points were widely scattered relative to the line of identity and demonstrated a low correlation coefficient (r^2^ = 0.13). Interestingly this analysis in RDN patients agrees closely with a recent report of consecutive office BP values in over 500,000 patients from a large clinical drug trial data set showing that the average magnitude of BP variation between 2 consecutive office visits was ±11.6 mmHg [30]. Another recent report of data from drug trials demonstrated that 24-h mean BP also exhibits visit to visit BP variability that is similar in magnitude to that for office BP. Importantly, office visit to visit variability was not correlated to 24 h BP visit to visit variability [31] which further complicates the definition of therapy responder. Furthermore, the variability in SBP is similar in magnitude to the actual population treatment effect reported in the sham controlled clinical trials. Therefore, the actual treatment effect within an individual cannot be distinguished from the variability effect when only a single type of measurement is used to define a responder. For example, even when the pretreatment BP is relying on two or more BP measurements performed at least 2–4 weeks apart, the individual BP response of less than 10.7 mmHg in the SPYRAL OFF and ON trial does not reliably identify a patient to be a responder to the specific intervention. Note that other factors such as antihypertensive drug non-adherence, psychosocial factors, and environmental influences [32], might also impact BP variability and hence the identification of true responders.

## Time response

**Fig. 4:**
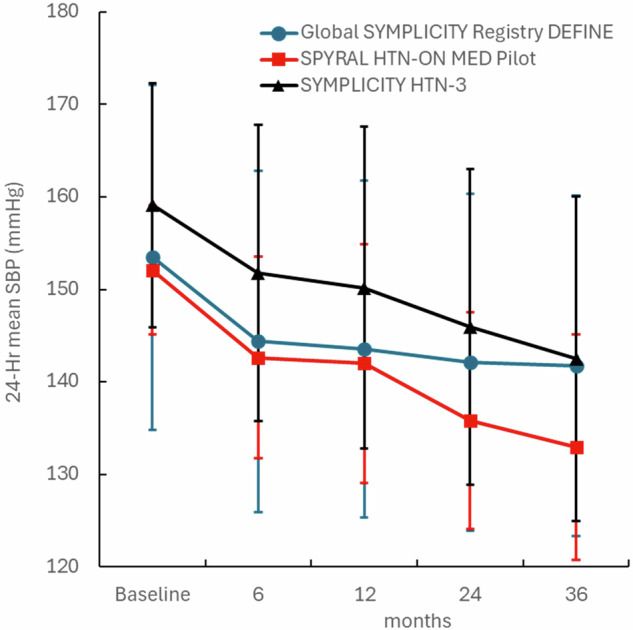
24-h systolic BP to 3 years in the Global SYMPLICITY Registry (blue, *N* = 2452), SPYRAL HTN-ON MED trial (red, *N* = 38); and SYMPLICITY HTN-3 trial (black, *N* = 360): (*P* < 0.05 vs. baseline for all time points using ANCOVA). Error bars are SD

Besides visit-to-visit BP variability, the changing response to the RDN procedure over time also complicates identification of procedural responders. Multiple recent reports have shown sequentially increased BP reductions over time that were not associated with increased medication burden. For example, 24-h blood pressure (Fig. 4) progressively decreased to 3 years post RDN in the Global SYMPLICITY Registry-DEFINE [33], SPYRAL HTN-ON MED pilot trial [34], and the SYMPLICITY HTN-3 trial [35] with minimal increase in prescribed antihypertensive medications. The mechanism for this progressive improvement in BP reduction is not yet clarified but may be related to recalibration of the neurohormonal cascade and reduction of vascular remodeling in the microcirculation known to need months and years for complete reversal [36]. Regardless of the mechanism, this increasing response to RDN over time suggests that any definition of “responders” must consider the timing of the assertion as well as the possibility that patients may change responder status over time when using traditional responder definitions.

One possible way to account for both visit to visit BP variability and increased response over time is to define responders using multiple time points. For example, “time in therapeutic target range” (TTR) is an estimate of the proportion of time spent within a specified BP range and has been shown to be an independent predictor of cardiovascular risk in hypertensive patients [37, 38]. Higher TTR within 6 months of RDN in the Global Symplicity-DEFINE registry was associated with fewer CV events, including stroke and death [39]. Additional analogous metrics of BP reduction that may also appropriately account for time include “hypertension burden” [40], time updated blood pressure [41], and “cumulative blood pressure load” [42].

## Wilder’s law of initial values

**Fig. 5:**
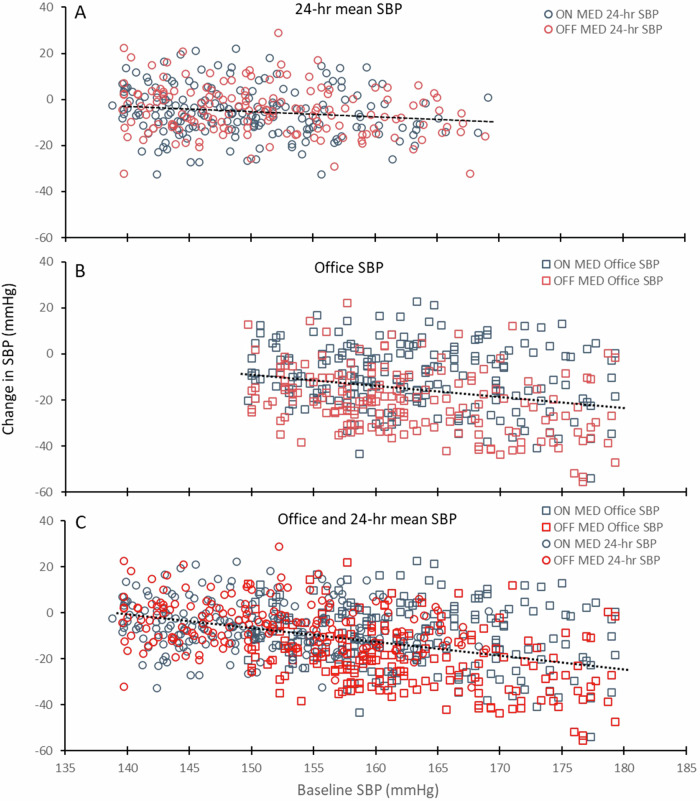
Reductions from baseline in 24-h mean and office SBP according to baseline BP following RDN in the SPYRAL HTN -OFF MED (blue, *N* = 182) and ON MED (red, *N* = 206) trials at the time of the primary endpoint (3 and 6 months, respectively). Dashed lines = linear regression fit. **A** 24 h-mean SBP (regression slope = −0.26; intercept = 33.6, r2 = 0.03; *p* = 0.001). **B** Office SBP (slope = −0.45; intercept = 57.5, r2 = 0.05; *p* < 0.001). **C** Both office (squares) and 24-h SBP (circles) SBP appear to follow a similar relationship (slope: −0.54; intercept = 73.9, r2 = 0.14; *p* < 0.001). Despite a significant correlation for all three regression fits, the relationship is weakly predictive

Wilder’s principle of initial value states that the response to a stimulus is related to the pre-stimulus level of the responding system [43, 44]. Considered from a practical perspective, those individuals with the greatest disease severity experience the greatest absolute and relative treatment benefit. This phenomenon is independent of other statistical phenomena such as regression to the mean and the Hawthorne effect, has been observed for several biomarkers including heart rate, LDL cholesterol or HBA1c, and has been applied to interventions for hypertension [45]. Analyses of office BP compared to 24-h BP showed a statistically significant linear relationship between the drop in office BP and the drop in ABPM from baseline following drug therapy [46, 47]. Accordingly, the change in BP following RDN is determined by the change in BP related to the baseline BP and the additional BP reduction related to RDN. The relationship between baseline BP and the subsequent change in BP at follow up for both office and 24-h SBP for all subjects randomized in the SPYRAL HTN -ON and -OFF MED trials is shown in Fig. 5. Note that the relationship between baseline SBP and SBP drop is comparable between 24-h (A) and office (B), when both parameters are plotted together (C). Interestingly, the increase in BP reduction at higher baseline BP was not observed in the sham group of the SPYRAL HTN-OFF MED trial as the average BP decrease was very low in the sham group.

Patients represented in the regression line may have no detectable treatment effect beyond the BP reduction related to the pretreatment BP level, or an apparent reverse effect, potentially due to changes in drug adherence behavior or to BP variability. Conversely, those patients represented below the regression line have a true BP lowering effect specifically related to the RDN intervention, independent from the nonspecific effect related to the baseline BP. Therefore, any definition of responder to a procedural intervention should also consider baseline BP and focus on the specific, baseline-independent BP decrease. For example, a recent single-center study of RDN in patients with and without CKD reported BP reductions in relation to baseline blood pressure that helped to identify similar responses in both groups [47].

## Conclusions and future directions

A useful definition of responders to interventions for BP must account for multiple factors including the method of BP measurement, visit to visit BP variability, increased treatment response over time and the baseline BP. Notably, these principles, exemplified here using data available from recent RDN trials, also fully apply to pharmacologic therapy, which also demonstrates variable response [46]. Combining office and 24-h BP parameters, including integration of multiple measurements, as well as assessing the true BP lowering effect of an intervention independent of unspecific response phenomenon, pairwise comparisons that consider drug changes as well as BP changes (generalized pairwise comparisons), may add insight into clinical trial results beyond those available when observing individual BP parameters in isolation. Future RDN trials should minimally consider longer term monitoring of home BP in order to allow for some of the suggested analyses. The current evidence may provide incentives for expert consensus statements [9, 48, 49] to accurately identify hypertension therapy responders.
